# Parasitism of Adult Pentatomidae by Tachinidae in Soybean in the North Central Region of the United States

**DOI:** 10.1093/jisesa/ieaa030

**Published:** 2020-05-04

**Authors:** Pheylan A Anderson, Daniela T Pezzini, Nádia M Bueno, Christina D DiFonzo, Deborah L Finke, Thomas E Hunt, Janet J Knodel, Christian H Krupke, Brian P McCornack, Christopher R Philips, Adam J Varenhorst, Robert J Wright, Robert L Koch

**Affiliations:** 1 Department of Entomology, University of Minnesota, St. Paul, MN; 2 Department of Entomology, Michigan State University, East Lansing, MI; 3 Division of Plant Sciences, University of Missouri-Columbia, Columbia, MO; 4 Department of Entomology, University of Nebraska, Concord, NE; 5 Department of Plant Pathology, North Dakota State University, Fargo, ND; 6 Department of Entomology, Purdue University, West Lafayette, IN; 7 Department of Entomology, Kansas State University, Manhattan, KS; 8 Department of Agronomy, Horticulture and Plant Science, South Dakota State University, Brookings, SD; 9 Department of Entomology, University of Nebraska-Lincoln, Lincoln, NE

**Keywords:** stink bug, biological control, parasitoid, Midwest, Diptera

## Abstract

Stink bugs (Hemiptera: Pentatomidae) are agricultural pests of increasing significance in the North Central Region of the United States, posing a threat to major crops such as soybean. Biological control can reduce the need for insecticides to manage these pests, but the parasitism of stink bugs by Tachinidae (Diptera) is poorly characterized in this region. The objective of this study was to evaluate the rate of parasitism of stink bugs by tachinids over 2 yr from nine states across the North Central Region. Parasitism was assessed by quantifying tachinid eggs on the integument of stink bug adults. Parasitism rates (i.e., percent of adult stink bugs with tachinid eggs) were compared across stink bug species, states, stink bug sex, and years. The mean percent parasitism of stink bugs by tachinids was about 6% across the region and did not differ among stink bug species. Mean percent parasitism was significantly higher in Missouri than in northern and western states. In addition, male stink bugs had significantly higher mean percent parasitism than females. Stink bug species commonly found in soybean in the region showed some parasitism and are therefore potentially vulnerable to oviposition by these parasitoids. This is the first study to characterize the level of parasitism of stink bugs by tachinids across the North Central Region.

Stink bugs (Hemiptera: Pentatomidae) are agricultural pests of increasing significance in the North Central Region of the United States (Koch et al. 2017). In soybean, *Glycine max* (L.) Merr. (Fabales: Fabaceae), stink bugs are of concern due to the increasing abundance of native species (Koch et al. 2017) and spread of the brown marmorated stink bug (*Halyomorpha halys* (Stål) [Hemiptera: Pentatomidae]) in the region (Rice et al. 2014, Pezzini et al. 2019). In soybean, stink bugs feed primarily on pods and seeds, and their injury is associated with, but not limited to, reduced seed quality and lower yield (Koch et al. 2017).

The primary response to stink bug outbreaks in most U.S. crops is the application of broad-spectrum insecticides, which may have negative impacts on the environment, natural enemy populations, and management programs for other pests (Rice et al. 2014). Identification of natural enemies, which can be used to enhance biological control, could lead to reduced insecticide use for management of these pests. Previous research on stink bug natural enemies in the North Central Region has focused mainly on those affecting egg masses of *H. halys* (e.g., Pezzini et al. 2018).

Tachinids are a diverse group of parasitoids with a wide variety of hosts and oviposition strategies to parasitize their hosts. The strategy utilized by tachinids that attack stink bugs involves ovipositing large macrotype eggs on the integument of the host, with the parasitoid larvae entering the host and consuming internal structures until the parasitoids eventually emerge and pupate (Stireman et al. 2006). Tachinids usually attack adult stink bugs and occasionally late-stage nymphs (Eger 1981, Kawada and Kitamura 1992, Francati et al. 2019). Several tachinids are reported to parasitize stink bug species. *Trichopoda pennipes* (F.) (Diptera: Tachinidae) is widely distributed, parasitizing stink bugs throughout the United States (Beard 1940, Pickett et al. 1996, Duncan 2017). Other tachinid species, such as *Gymnosoma par* (Walker), *Gymnoclytia occidua* (Walker), *Euthera tentatrix* Loew, *Euclytia flava* (Townsend), and *Cylindromyia fumipennis* (Bigot) (Diptera: Tachinidae), are known to parasitize native stink bugs and are present in the North Central Region (Aldrich et al. 2006, Duncan 2017).

Data on the parasitism of stink bugs by tachinids is limited in the North Central Region. This study was intended to quantify tachinid parasitism of stink bug species collected in soybean in the region. Over a 2-yr period, stink bugs were collected from multiple fields across nine states and examined for tachinid eggs, resulting in a comparison of parasitism by stink bug species, state, sex, and year. Results of this study will inform further research evaluating the impact of tachinids as potential biological control agents of stink bugs.

## Methods

### Sampling Locations

In 2016 and 2017, stink bugs were collected from soybean fields on university research stations and commercial farms in nine states of the North Central Region. These stink bugs were collected for another study investigating community structure and abundance of stink bugs in soybean and could be used for this study due to the large quantity of intact adults that were saved (Pezzini et al. 2019). In 2016, 50 fields were sampled from sites in Indiana, Kansas, Minnesota, Missouri, Nebraska, North Dakota, and South Dakota. In 2017, 51 fields were sampled from sites in Indiana, Michigan, Minnesota, Missouri, Nebraska, North Dakota, Ohio, and South Dakota. Within each state, there were one to four fields per site and up to four sites per state. These sites were 13–368 km apart within states. The locations of sites at which fields were located are depicted in Figure 1 of Pezzini et al. (2019). Field sizes ranged from 0.5 to 120 ha (mean ± SEM: 17.9 ± 2.1 ha) with 76.2 cm row spacing. Soybean varieties, planting dates and management varied according to the common practices of each state, as indicated in Pezzini et al. (2019).

### Sampling Method

Each field was sampled using a 39 cm diameter sweep net to collect sample units of 25 sweeps. At least 12 sample units were collected from each field on every sample date. In each year, fields were sampled on at least four different dates beginning mid-July up to early October, which generally corresponded to beginning bloom (R1) through full maturity (R8) soybean growth stages (Fehr and Caviness 1977). The contents of each 25-sweep sample unit were placed in a 20.3 × 25.4 cm zippered plastic bag. Bags were stored in a freezer until processed. In laboratories in each state, sample contents were carefully sorted to remove plant material and other insects. Samples were then sent to the University of Minnesota, where adult stink bugs were pinned, identified to species, and kept in insect boxes at room temperature for later assessment of parasitism by tachinids. Adult stink bug species were identified based on McPherson and McPherson (2000), Rider (2012), and Paiero et al. (2013).

### Assessment of Parasitism

Because these stink bugs were collected as part of another study focused on characterization of this community (Pezzini et al. 2019), they could not be maintained alive to rear out adult tachinids. Therefore, parasitism was assessed by visual inspection for tachinid eggs on the integument of the pinned stink bug specimens. The entire body surface of each stink bug was examined using fine-tipped forceps under a dissecting microscope. Such visual assessment of parasitism is a common method that has been validated by previous research (Eger 1981, Harris and Todd 1981, McPherson et al. 1982, Kawada and Kitamura 1992, Agostinetto et al. 2018). The number of eggs on each stink bug was recorded. Eggs were confirmed to be Tachinidae by visual examination of high-resolution photographs of the eggs by Dr. John Stireman III, Wright State University. Voucher specimens have been placed in the University of Minnesota Insect Collection.

### Data Analyses

Analyses were conducted with R version 3.4.4 (R Core Team 2018). Rate of parasitism was calculated as the proportion of individual stink bugs with at least one tachinid egg on their body surface. Because of complete separation in the data, the effects of stink bug species, state, sex, year, and their interactions on parasitism rate were tested as a binomial response using bias-reduced generalized linear models (BRGLM) (package: brglm2; Kosmidis 2019). Through backward model selection, interactions were found to be nonsignificant and were removed from the model. Analyses of significant differences among states were determined using Tukey–Kramer adjusted pairwise comparisons of least square means at α = 0.05 (package: multcomp; Hothorn et al. 2008).

## Results

**Fig. 1.**
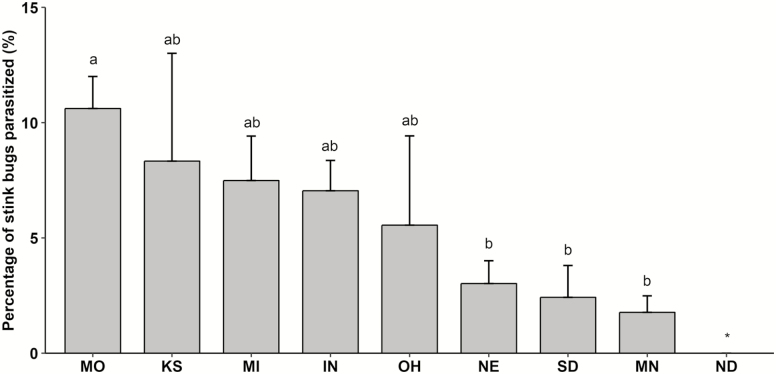
Mean (±SEM) percentage tachinid parasitism for a total of 1,968 stink bugs collected in soybean sweep samples from nine states in the North Central Region of the United States. Means with same letters are not significantly different at α = 0.05 using Tukey–Kramer-adjusted pairwise comparisons of least square means. An asterisk (*) indicates no parasitism observed for that state (IN = Indiana, KS = Kansas, MI = Michigan, MN = Minnesota, MO = Missouri, NE = Nebraska, ND = North Dakota, OH = Ohio, SD = South Dakota).

A total of 1,968 stink bug adults (980 males and 959 females) were collected across states and years (Pezzini et al. 2019). Among these individuals, 6.0% were parasitized (i.e., had at least one tachinid egg on their body surface). Mean percent parasitism ranged from 1.6 to 16.0% among the eight stink bug species with parasitism, but did not differ significantly (χ ^2^ = 4.374, df = 7, *P* = 0.735) (Table 1). An additional eight stink bug species were collected at low abundance in the survey and showed no evidence of parasitism by tachinids (Table 1). Mean percent parasitism of stink bugs differed significantly among states (χ ^2^ = 21.799, df = 8, *P* = 0.005) with parasitism in Missouri being 3.5 to 5.9× greater than in Nebraska, South Dakota and Minnesota (Fig. 1). No parasitism was observed in North Dakota throughout the study. In addition, mean percent parasitism of stink bugs differed significantly between sexes (χ ^2^ = 16.061, df = 1, *P* < 0.001) with parasitism of males being 2.2× greater than that of females. Furthermore, mean percent parasitism of stink bugs differed significantly between years (χ ^2^ = 4.885, df = 1, *P* = 0.027), with parasitism in 2017 being 1.4× greater than in 2016. Parasitism rates were 6.7 ± 0.9 and 3.6 ± 0.7 for males and females in 2016, and 10.7 ± 1.6 and 3.9 ± 0.9 for males and females in 2017.

**Table 1.** Tachinid parasitism of stink bugs collected from soybean across nine states in the North Central Region of the United States in 2016 and 2017

Species^*a*^	Number of individuals	Percent parasitism
*Chinavia hilaris*	683	9.4
*Euschistus servus* ^*b*^	125	7.2
*Euschistus tristigmus* ^*c*^	61	1.6
*Euschistus variolarius*	871	3.7
*Halyomorpha halys*	31	12.9
*Podisus maculiventris*	106	2.8
*Thyanta calceata*	6	16.7
*Thyanta custator accerra*	59	5.1

^*a*^Species collected in survey, but not parasitized by tachinids were *Banasa dimidiata* (Say) (*n* = 1), *Coenus delius* (Say) (*n* = 2), *Cosmopepla lintneriana* Kirkaldy (*n* = 8), *Holcostethus limbolarius* (Stål) (*n* = 3), *Mormidea lugens* (Fabricius) (*n* = 1), *Oebalus pugnax* (Fabricius) (*n* = 8), *Trichopepla semivittata* (Say) (*n* = 1), and *Apoecilus cynicus* (Say) (*n* = 2).

^*b*^Includes *E. servus servus* (Say), *E. servus euschistoides* (Vollenhoven), and the hybrid.

^*c*^Includes *E. tristigmus luridus* Dallas and *E. tristigmus tristigmus* (Say).

In total, 218 tachinid eggs were observed. The mean number of tachinid eggs per stink bug was 1.9, with a range of 1 to 12 eggs per stink bug. Of the parasitized individuals, the majority (59%) had one egg, followed by 20% with two eggs, 8% with three eggs and 13% with 4–12 eggs.

## Discussion

This is the first study to examine tachinid parasitism of stink bugs in soybean in the North Central Region. Our results show that the rate of parasitism of stink bug species by tachinids was relatively low in this region (6.0%). Even though our study was limited to stink bugs collected from soybean fields, the average rate of parasitism reported here is similar to that of Duncan (2017) who sampled stink bugs from different habitats in Ohio. Therefore, it appears that tachinids may not contribute substantially to natural control of stink bugs in the region.

Eight stink bug species, which comprised >98% of the stink bugs collected from soybean in the North Central Region (Pezzini et al. 2019), were parasitized by tachinids in our study. This suggests that the majority of stink bugs present in the crop are vulnerable to oviposition by tachinids. We found no significant difference in parasitism among the eight species, including the invasive *H. halys* (Table 1). Similarly, Duncan (2017) reported no significant difference in parasitism between *H. halys* and native species in Ohio. The effect of parasitism by tachinids on *H. halys* populations is unclear, as Rice et al. (2014) noted mean parasitism rates of 1–5%, but negligible emergence of adult tachinids. *Halyomorpha halys* may act as an egg sink for some tachinid species (Abram et al. 2014), which could adversely affect the biological control they provide. 

Our results showed parasitism rates differed significantly among states (Fig. 1). The regional pattern of stink bug parasitism may be due to a combination of biotic and abiotic factors that may vary across states in the region. Pezzini et al. (2019) showed a similar geographic gradient in the abundance of stink bugs from which these tachinid eggs were quantified, with abundance generally decreasing from southeast to northwest. It is possible that areas with greater abundance of stink bugs support a greater abundance or diversity of tachinids. Furthermore, landscape composition, climatic condition and crop management are known to affect natural enemy populations and the biological control they provide (Gardiner et al. 2009, Zerbino and Panizzi 2019). For example, availability of nectar-producing plants increased parasitism of *Nezara viridula* (Linnaeus) (Hemiptera: Pentatomidae) by *T. pennipes* in Georgia (Tillman and Carpenter 2014). In addition, areas with reduced insecticide applications have greater parasitism of stink bugs (Ferreira Santos de Aquino et al. 2019).

We found that male stink bugs were parasitized more than females, which agrees with previous literature. Studies on *N. viridula* in Hawaii (Mitchell and Mau 1971) and Louisiana (McPherson et al. 1982), as well as a study of *Podisus maculiventris* (Say) (Hemiptera: Pentatomidae) in Maryland (Aldrich et al. 1984), also found greater rates of parasitism of males. Unlike many insect species, male production of sex pheromones is common among stink bugs. For example, the males of *Euschistus conspersus* Uhler (Hemiptera: Pentatomidae), a species native to the western United States, produce an aggregation pheromone (Krupke et al. 2001) that attracts ovipositing tachinids as well (Krupke and Brunner 2003).

Overall, parasitism of adult stink bugs by tachinids in our survey of the North Central Region was relatively low, indicating that tachinids may be relatively minor contributors to natural control of stink bugs in the region. However, since the most abundant stink bug species collected in soybean each showed some level of parasitism by tachinids, these common species are apparently vulnerable to attack by tachinids. Further work could examine how to enhance these tachinid populations and the biological control they offer (e.g., Tillman and Carpenter 2014, Zerbino and Panizzi 2019). In addition, future work could be informed by rearing adult tachinids from stink bug hosts to determine the tachinid species parasitizing stink bugs in this region.
